# RNAi Screening Identifies that TEX10 Promotes the Proliferation of Colorectal Cancer Cells by Increasing NF‐*κ*B Activation

**DOI:** 10.1002/advs.202000593

**Published:** 2020-07-07

**Authors:** Ziyang Wang, Chunjie Sheng, Guangyan Kan, Chen Yao, Rong Geng, Shuai Chen

**Affiliations:** ^1^ Sun Yat‐sen University Cancer Center State Key Laboratory of Oncology in South China Collaborative Innovation Center for Cancer Medicine Guangzhou Guangdong 510060 P. R. China

**Keywords:** colorectal cancer, proliferation, RELA, RNAi screening, TEX10

## Abstract

Colorectal cancer (CRC) has become a predominant cancer worldwide. To understand the process of carcinogenesis, a short hairpin RNA library screening is employed to search for candidate genes that promote proliferation in the CRC cell line HT29. The candidate genes overlap with differentially expressed genes in 32 CRC tumor tissues in the GEO dataset GSE8671. The seventh‐ranked testis expressed 10 (TEX10) is upregulated in CRC and its knockdown decreases cell proliferation. The TEX10 high‐expression group exhibits worse overall survival (*P *= 0.003) and progression‐free survival (*P =* 0.001) than the TEX10 low‐expression group. TEX10 depletion decreases the growth of CRC cells in vitro and in vivo. Gene set enrichment analysis indicates that the nuclear factor‐kappa B pathway is significantly enriched in the genes downregulated by TEX10 knockdown. Mechanistically, TEX10 interacts with RELA and increases its nuclear localization. TEX10 promotes RELA occupancy at gene promoters and regulates the expression of a subset of RELA‐targeted genes, including *TNFAIP8*, *SAT1*, and *IL6ST*. Taken together, this study identifies that TEX10 promotes the proliferation of CRC cells in an RELA‐dependent manner. In addition, high TEX10 expression is associated with poor prognosis in CRC patients.

## Graphical abstract

This study performs genome‐wide RNA interference (RNAi) screening to identify testis expressed 10 (TEX10) as an essential gene in colorectal cancer (CRC). Upregulation of TEX10 in CRC is associated with poor prognosis of patients. TEX10 interacts with RELA and increases its nuclear localization, therefore stimulating the expression of a subset of RELA‐targeted genes. TEX10 may serve as a valuable biomarker and therapeutic target in CRC.

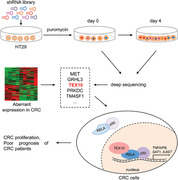

## 1 Introduction

Colorectal cancer (CRC) has become a predominant cancer worldwide and its incidence and mortality have been high for many years.^[^ ^1^ ^]^ In the past 30 years, the molecular mechanism of CRC has been increasingly understood, which remains very important for the treatment of CRC.^[^ ^2^, ^3^ ^]^ Many genes, such as the oncogenes *EGFR*, *CMYC*, and *KRAS*, are abnormally expressed or mutated in CRC, thus promoting the proliferation, metastasis, and progression of CRC.^[^ ^4^ ^]^ The most fundamental characteristic of cancer cells is their sustained and rapid proliferation.^[^ ^5^ ^]^ In tumors, abnormal activation of the nuclear factor‐kappa B (NF‐*κ*B) signaling pathway provides a continuous signal for tumor proliferation.^[^ ^6^ ^]^

NF‐*κ*B is a transcription factor complex that includes RELA (also known as p65), RELB, Rel, NF‐*κ*B1 p50/p105, and NF‐*κ*B2 p52/p100.^[^ ^7^ ^]^ The most abundant form of these NF‐*κ*B proteins is RELA complexed with p50.^[^ ^8^ ^]^ In unstimulated cells, the RELA‐p50 complex binds to the inhibitory protein NF‐*κ*B inhibitor alpha (I*κ*B*α*) and is thus retained in the cytoplasm.^[^ ^9^ ^]^ Upon cellular stimulation by proinflammatory cytokines such as tumor necrosis factor (TNF), the TNF receptor transmits the stimulus, leading to the ubiquitination of I*κ*B*α*.^[^ ^10^ ^]^ Ubiquitinated I*κ*B*α* is degraded, allowing the RELA‐p50 complex to enter the nucleus.^[^ ^11^ ^]^ In the nucleus, NF‐*κ*B transcription factors bind to promoters or enhancer sites on target genes and regulate the expression of genes involved in various biological functions, such as inflammation, development and cell growth.^[^ ^12^ ^]^ In normal cells, activation of NF‐*κ*B family transcription factors is strictly regulated and inappropriate activation of NF‐*κ*B has been linked to various cancers.^[^ ^13^ ^]^ Among these links, the relationship between constitutively activated NF‐*κ*B signaling and accelerated tumor progression and poor prognosis in CRC has been widely studied and reported.^[^ ^14^, ^15^ ^]^

In the present study, we performed a short hairpin RNA (shRNA) library screening and identified testis expressed 10 (*TEX10*) as an essential gene in CRC cells. A previous study has shown that Tex10 is enriched at super‐enhancers in an Sox2‐dependent manner and plays a key role in embryonic stem cell self‐renewal.^[^ ^16^ ^]^ Here, we report that TEX10 is highly expressed in CRC and is associated with a poor prognosis of CRC. TEX10 promotes the proliferation of CRC cells by binding to RELA and increasing its nuclear localization. These results indicate that *TEX10* functions as an oncogene and a valuable biomarker in CRC and may thus be a therapeutic target in CRC patients.

## 2 Results

### 2.1 Screening Indicates That TEX10 Is Upregulated and Is Associated with Prognosis in CRC

**Figure 1:**
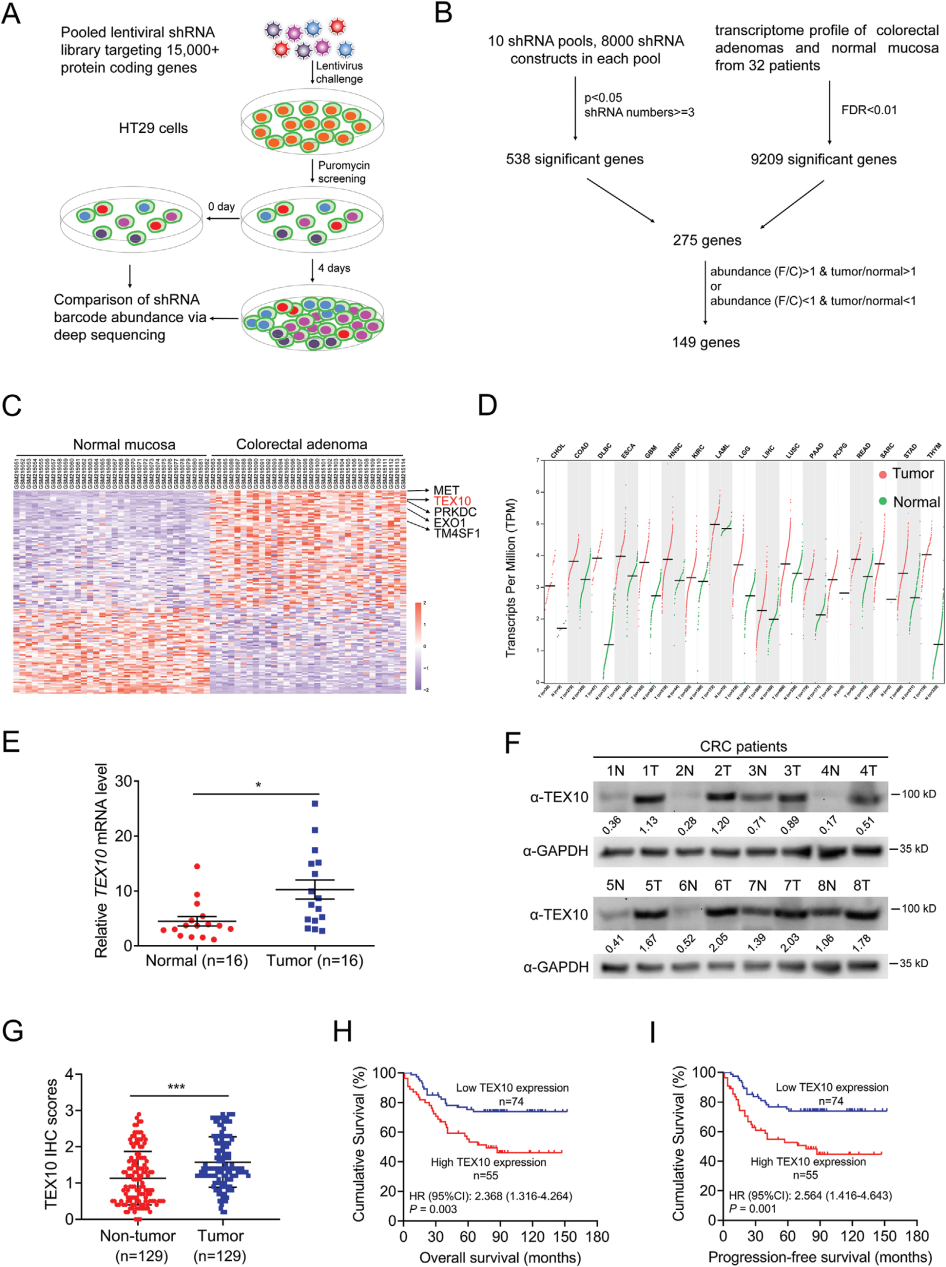
Screening indicates that TEX10 is upregulated and is associated with prognosis in CRC. A) Schematic of the shRNA library screening strategy for genes that affected CRC cell proliferation. B) Schematic of the overlapping results of shRNA‐screened genes and differentially expressed genes in tumors. C) Heatmap of the overlapping genes in (B). D) TEX10 expression profile in various tumor types and normal tissues as identified through the GEPIA website. E) qPCR analysis of *TEX10* expression in 16 CRC tissues and adjacent normal mucosal tissues. F) IB analysis of TEX10 protein expression in paired CRC tissues. N, normal tissues; T, tumor tissues. G) IHC scores of TEX10 in 129 paired CRC and adjacent normal tissues. H) Kaplan–Meier analysis of overall survival and I) progression‐free survival according to TEX10 levels in CRC patients. *P < 0.05, **P < 0.01, and ***P < 0.001 (E,G) two‐tailed Student's *t*‐test or H,I) log‐rank test).

To identify genes that promote the proliferation of CRC, we designed an shRNA‐based screening strategy^[^ ^17^ ^]^ in HT29 cells (**Figure** **1**A,B). By calculating the abundance of each shRNA sequence in day 0 and day 4 cells, we focused on 538 genes targeted by 1808 shRNAs that met the following criteria: 1) the abundance of the shRNA against the gene in day 0 cells was greater than 25 copies per million sequences; 2) the difference in the shRNA abundance between the day 0 group and the day 4 group had a *P* of <0.05; and 3) the number of shRNAs targeting a single gene was ≥3. These 538 genes further overlapped with 275 genes that were differentially expressed in CRC tissues (Gene Expression Omnibus (GEO) dataset GSE8671;^[^ ^18^ ^]^ Figure 1B). We next selected genes that promoted cell growth and exhibited increased expression in CRC tissues, or those that inhibited cell growth and exhibited decreased expression in CRC tissues, and then ranked the resulting 149 genes according to their false discovery rate (FDR) values (Figure 1B,C). These genes were distributed in multiple pathways (Figure S1A, Supporting Information), and some of them, such as *MET* ^[^ ^19^ ^]^ (ranked 1st), *PRKDC* ^[^ ^20^ ^]^ (ranked 8th), *EXO1* ^[^ ^21^ ^]^ (ranked 14th), and *TM4SF1* ^[^ ^22^ ^]^ (ranked 25th), have been reported to promote tumor cell proliferation (Figure 1C). Among the top nine genes (Table S1, Supporting Information), five genes have been reported to affect CRC progression (*MET*,^[^ ^19^ ^]^ *DKC1*,^[^ ^23^ ^]^ *GRHL3*,^[^ ^24^ ^]^ *PRKDC*,^[^ ^20^ ^]^ and *GPC4* ^[^ ^25^ ^]^). We individually silenced the remaining four genes (*WER75*, *FAIM*, *GCSH*, and *TEX10*) with two independent synthesized small interfering RNAs (siRNAs) in HT29 cells (Figure S1B, Supporting Information) and we found that the knockdown of *TEX10*, which ranked 7th in the aberrant expression analysis and was identified as an essential gene in the shRNA library screening (Table S1, Supporting Information), had the highest inhibitory efficacy on the growth of HT29 cells (Figure S1C, Supporting Information). We then chose to study the function of *TEX10* in CRC.

As overexpression of TEX10 in CRC and other tumor types was supported by the Cancer Genome Atlas (TCGA) database (Figure 1D), we performed quantitative real‐time polymerase chain reaction (PCR) (qPCR; Figure 1E), immunoblot (IB; Figure 1F), and immunohistochemistry (IHC; Figure 1G and Figure S1D, Supporting Information) analyses to identify higher TEX10 levels in CRC tissues than in paired normal tissues. Kaplan–Meier survival analysis demonstrated that CRC patients with high TEX10 levels had worse overall survival (OS, *P *= 0.003) and progression‐free survival (PFS, *P =* 0.001) than those with low levels (Figure 1H,I), which coincides well with the data from the GEO datasets (Figure S1E, Supporting Information). Multivariate Cox proportional hazards regression analysis revealed that the TEX10 level, together with the tumor invasion depth, lymph node status, distant metastasis status, stage, and preoperative CA199 level were significantly correlated with both overall survival and progression‐free survival (Table S2, Supporting Information). Multivariate analysis of these parameters indicated that TEX10 expression was an independent prognostic biomarker (Table S3, Supporting Information). Taken together, we identified TEX10 as an essential gene in CRC and its high expression was associated with a poor prognosis in CRC patients.

### 2.2 TEX10 Depletion Suppresses the Growth of CRC Cells In Vitro

**Figure 2:**
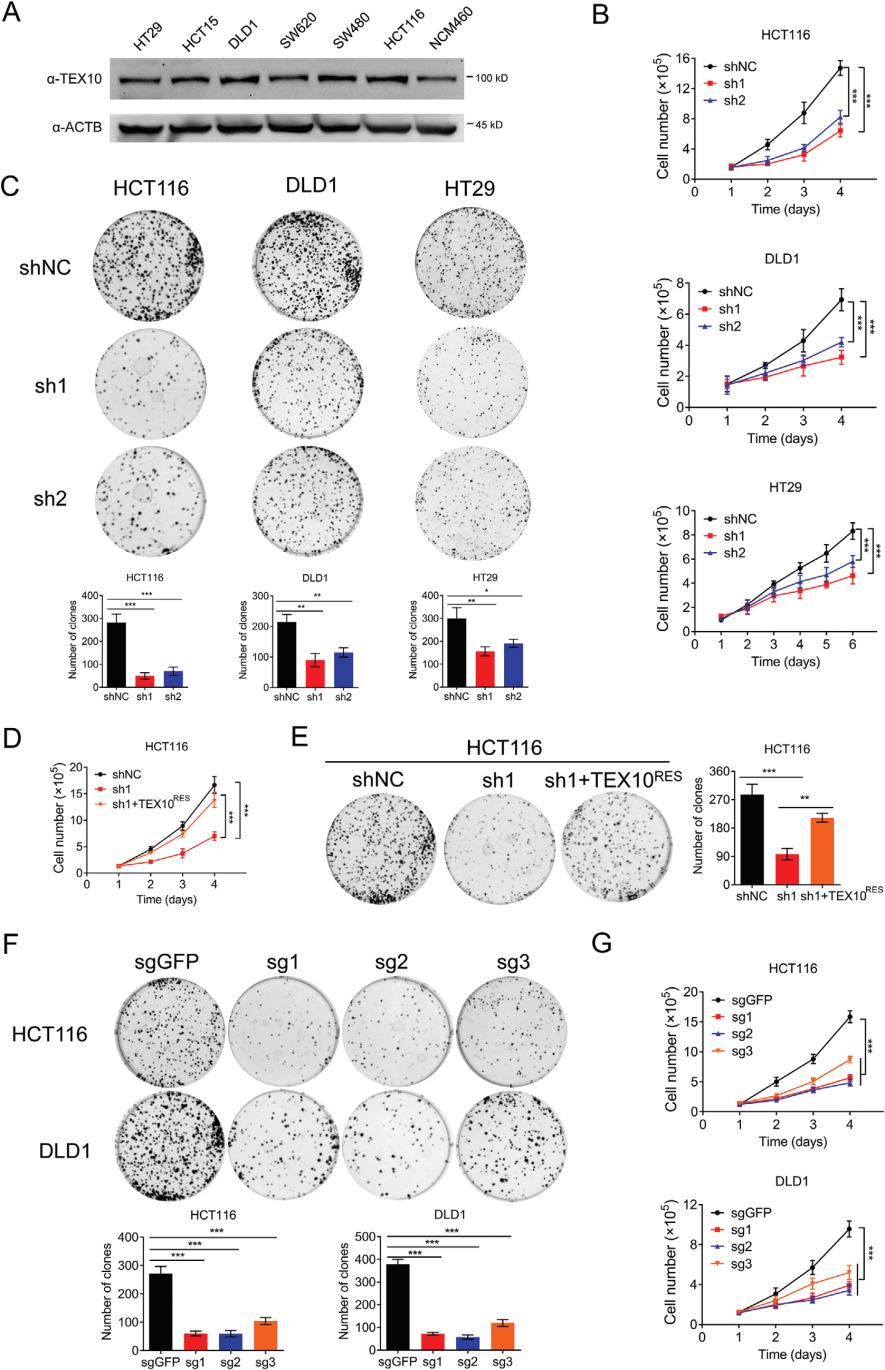
Depletion of TEX10 suppresses the growth of CRC cells in vitro. A) IB analysis of endogenous TEX10 expression in CRC cell lines. B) Cell count analysis and C) colony formation assay of the growth of shNC and TEX10 knockdown cells in the HCT116, DLD1, and HT29 cells. D) Cell count analysis and E) colony formation assay of the growth of HCT116 shNC, TEX10‐sh1, and TEX10‐sh1 cells reconstituted with TEX10^RES^. F) Colony formation assay and G) cell count analysis of HCT116 and DLD1 control and TEX10 knockout cells. *P < 0.05, **P < 0.01, and ***P < 0.001 (B,E,F) one‐way ANOVA with Bonferroni's post‐test or C,D,G) one‐way ANOVA with Bonferroni's post‐test).

As TEX10 was highly expressed in HCT116 and DLD1 cells (**Figure** **2**A and Figure S2A, Supporting Information), we designed two independent shRNAs that targeted TEX10 and differed from the five shRNAs in the shRNA library to knockdown this gene in HCT116 and DLD1 cells as well as HT29 cells (Figure S2B, Supporting Information). The cell count analysis (Figure 2B), colony formation assay (Figure 2C), and 5‐ethynyl‐2’‐deoxyuridine (EdU) staining (Figure S2C, Supporting Information) results showed that TEX10 knockdown significantly reduced the growth of these CRC cells. Subsequently, we synonymously mutated TEX10 wild‐type cDNA to an shRNA‐1‐resistant TEX10 that encoded the same protein (indicated as TEX10^RES^). We exogenously expressed TEX10^RES^ in HCT116‐sh1 cells (Figure S2D, Supporting Information). TEX10 knockdown decreased cell proliferation and this phenotype was significantly rescued by TEX10^RES^ overexpression (Figure 2D,E and Figure S2E, Supporting Information). Moreover, TEX10 knockout using CRISPR‐Cas9 technology (Figure S2F, Supporting Information) also decreased the growth of HCT116 and DLD1 cells (Figure 2F,G). These results indicated that TEX10 depletion decreased the growth of CRC cells in vitro.

### 2.3 TEX10 Depletion Reduces NF‐*κ*B Activity

**Figure 3:**
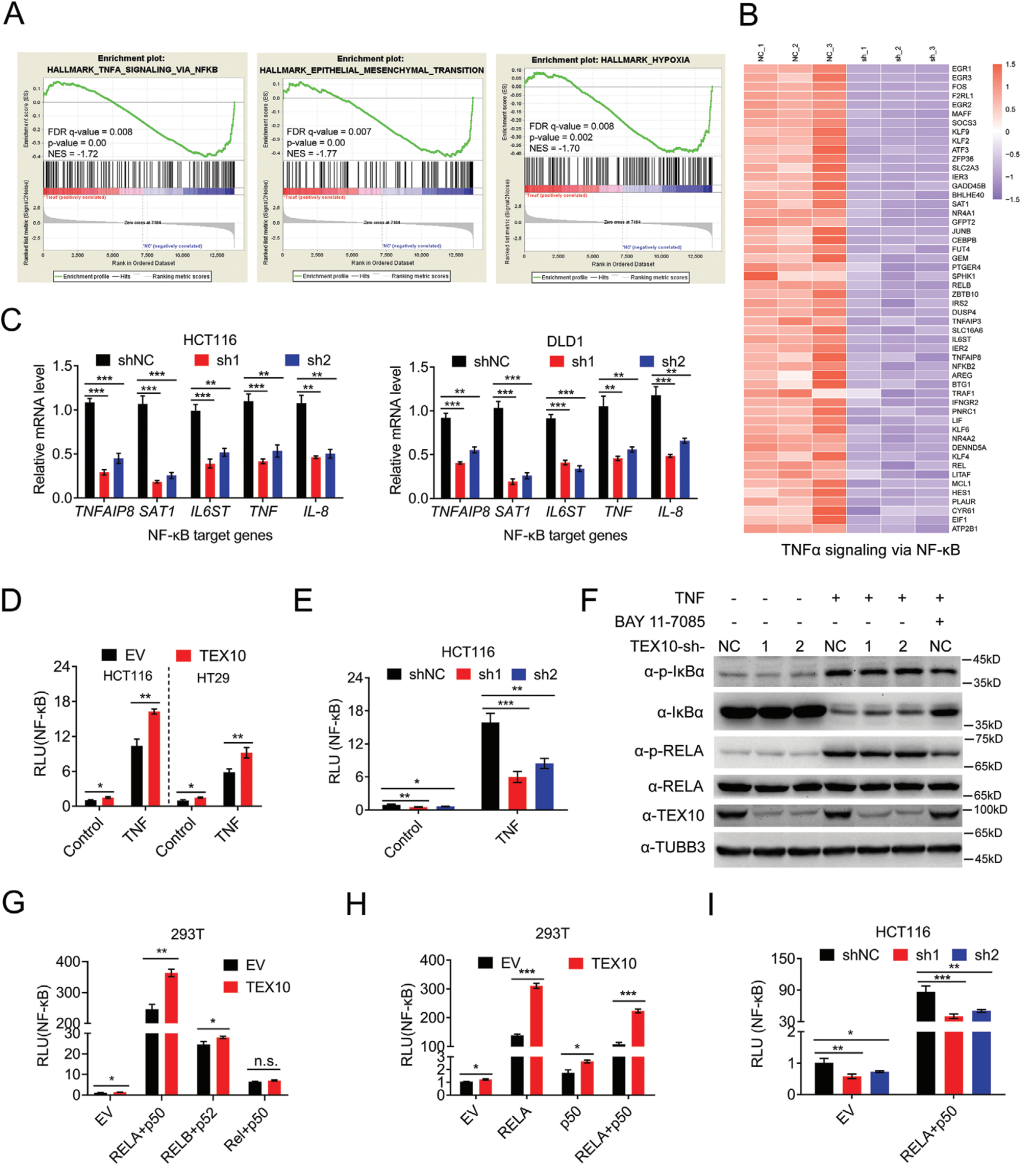
TEX10 depletion reduces NF‐*κ*B activity. A) Gene set enrichment analysis (GSEA) of the RNAseq results of TEX10 knockdown and shNC HCT116 cells. B) Heatmap of TEX10 knockdown‐regulated genes involved in the TNF–NF‐*κ*B pathway. C) qPCR analysis of the mRNA expression of the indicated NF‐*κ*B‐targeted genes in TEX10 knockdown and shNC HCT116 and DLD1 cells. D) Luciferase assay of NF‐*κ*B activity in HCT116 and HT29 cells transfected with empty vector (EV) or TEX10 and then treated with TNF for 6 h. E) Luciferase assay of NF‐*κ*B activity in TEX10 knockdown and shNC HCT116 cells treated with TNF for 6 h. F) IB analysis of phosphorylated I*κ*B*α* (p‐I*κ*B*α*), total I*κ*B*α*, phosphorylated RELA (p‐RELA), total RELA, TEX10, and TUBB3 in TEX10 knockdown and shNC HCT116 cells treatment with TNF or NK‐*κ*B inhibitor BAY 11‐7085. G) Luciferase assay of NF‐*κ*B activity in HEK293T cells transfected with EV or with TEX10 together with EV, RELA plus p50, RELB plus p52, or Rel plus p50 or H) combinations of EV, RELA, p50 and RELA plus p50 for 24 h. I) Luciferase assay of NF‐*κ*B activity in TEX10 knockdown or shNC HCT116 cells transfected with EV or RELA plus p50. *P < 0.05, **P < 0.01, and ***P < 0.001 (D,G,H) two‐tailed Student's *t*‐test or C,E,I) one‐way ANOVA with Bonferroni's post‐test).

To elucidate the molecular mechanisms by which TEX10 promotes CRC cell proliferation, we performed RNA sequencing (RNAseq) analysis in TEX10 knockdown HCT116 cells. Gene set enrichment analysis (GSEA) demonstrated that the TNF–NF‐*κ*B pathway, epithelial‐to‐mesenchymal transition (EMT) pathway and hypoxia response genes were significantly enriched in the downregulated genes (**Figure** **3**A). NF‐*κ*B target genes with decreased expression as determined by RNAseq are shown in Figure 3B and included *TNFAIP8*, *SAT1*, and *IL6ST*. In addition, our qPCR analysis results confirmed that TEX10 depletion decreased the expression of *TNFAIP8*, *SAT1*, *IL6ST*, and the canonical NF‐*κ*B inducer genes *TNF* and *IL‐8* in HCT116 and DLD1 cells (Figure 3C). TEX10 overexpression promoted the expression of these genes in a dose‐dependent manner in HT29 cells (Figure S3A–C, Supporting Information). The dual‐luciferase assay^[^ ^26^ ^]^ results showed that TEX10 overexpression significantly promoted NF‐*κ*B activity in HCT116 and HT29 cells (Figure 3D), whereas TEX10 knockdown produced the opposite phenomenon in HCT116 cells (Figure 3E).

Phosphorylation and degradation of I*κ*B*α* and phosphorylation of RELA are the crucial steps in NF‐*κ*B activation.^[^ ^27^ ^]^ Depletion of *TEX10* did not affect I*κ*B*α* phosphorylation and degradation or RELA phosphorylation (Figure 3F). Considering that TEX10 accumulates dominantly in the nucleus,^[^ ^16^ ^]^ this phenomenon suggests that TEX10 directly regulates NF‐*κ*B transcription factors without affecting the upstream signaling axis. Then, we examined the effect of TEX10 on NF‐*κ*B proteins and found that TEX10 overexpression significantly enhanced RELA and p50‐induced NF‐*κ*B compared with other protein‐induced NF‐*κ*B activity (Figure 3G). The NF‐*κ*B proteins RELA and p50 are transcription factors in the canonical NF‐*κ*B pathway,^[^ ^8^ ^]^ and among these two transcription factors, TEX10 has a stronger effect on RELA (Figure 3H). TEX10 knockdown significantly inhibited NF‐*κ*B activity induced by RELA and p50 in HCT116 cells (Figure 3I). Moreover, TNF treatment did not affect TEX10 expression in HCT116 and DLD1 cells (Figure S3D, Supporting Information). Based on these results, we concluded that TEX10 is a stimulator of NF‐*κ*B signal pathway.

### 2.4 TEX10 Interacts with the RELA N‐Terminal Region

**Figure 4:**
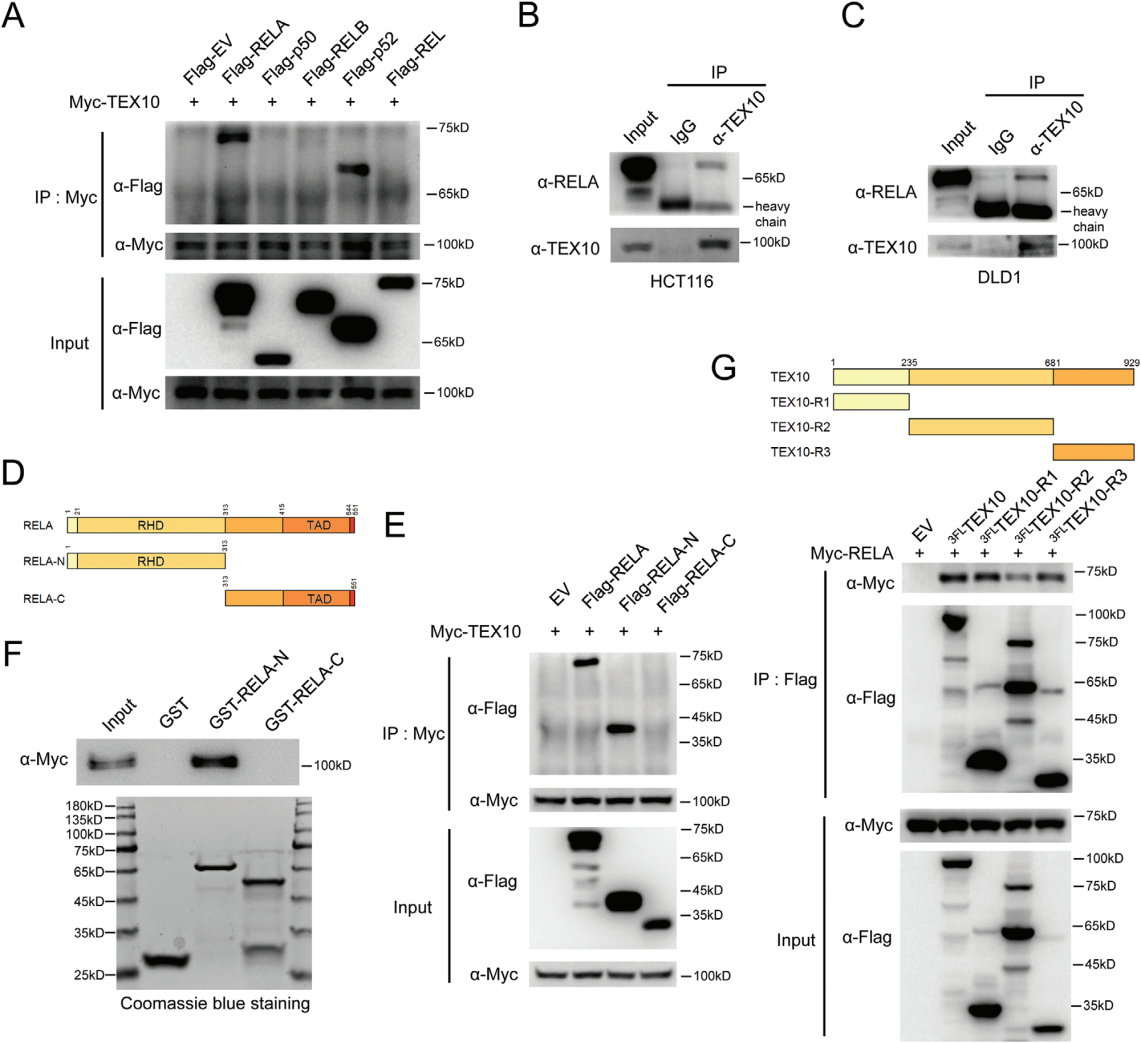
TEX10 interacts with the RELA N‐terminal region. A) Lysates from HEK293T cells cotransfected with plasmids expressing Flag‐tagged EV, RELA, p50, RELB, p52, or REL along with Myc‐TEX10 were immunoprecipitated with an anti‐Myc antibody (*α*‐Myc) and subjected to IB analysis with an anti‐Flag antibody (*α*‐Flag) and *α*‐Myc. Lysates from B) HCT116 and C) DLD1 cells were immunoprecipitated with immunoglobulin G (IgG) or an anti‐TEX10 antibody (*α*‐TEX10) and subjected to IB analysis with an anti‐RELA antibody (*α*‐RELA) and *α*‐TEX10. D) Schematic diagram of RELA and its truncation mutants. RHD, Rel homology domain; TAD, transactivation domain. E) Lysates from HEK293T cells cotransfected with plasmids for Flag‐tagged EV, RELA, RELA‐N, or RELA‐C along with Myc‐TEX10 were immunoprecipitated with *α*‐Myc and subjected to IB analysis with *α*‐Flag and *α*‐Myc. F) GST pulldown assay of the binding of in vitro‐translated Myc‐TEX10 with GST and the GST‐RELA‐N and GST‐RELA‐C truncations. G) Schematic diagram of TEX10 and its recombinant protein truncation mutants (top). Lysates from HEK293T cells cotransfected with plasmids expressing 3Flag‐tagged EV, TEX10, or TEX10 truncations along with Myc‐RELA were immunoprecipitated with *α*‐Flag and subjected to IB analysis with *α*‐Myc and *α*‐Flag (bottom).

We performed coimmunoprecipitation (CoIP) assays to investigate the binding of TEX10 to NF‐*κ*B proteins. Ectopically expressed TEX10 interacted with RELA and p52 in cells (**Figure** **4**A). Considering that the function of RELA was significantly stronger than p52 to stimulate NF‐kB activity in combination with TEX10 enforced expression (Figure 3G) and *RELA* was highly expressed in colon while *NFKB2* (the precursor protein of p52) was mainly expressed in lymphocytes^[^ ^28^ ^]^ (Figure S4, Supporting Information), we next focused on RELA and confirmed the endogenous interaction between TEX10 and RELA in HCT116 and DLD1 cells (Figure 4B,C). To narrow down the regions in both proteins that control their interaction, we generated RELA truncations, including those with only the Rel homology domain (RHD) or the transcription activation domain (TAD) (Figure 4D), and TEX10 truncations, including region 1 (R1, amino acid (A.A.). 1–235), region2 (R2, A.A. 236–681), and region 3 (R3, A.A. 682–929) truncations (Figure 4G). The CoIP assay revealed that the RHD domain but not the TAD domain of RELA specifically formed a complex with TEX10 (Figure 4E), and this result was further confirmed in the glutathione S‐transferase (GST) pulldown assay, in which in vitro‐translated TEX10 directly interacted with the RELA RHD domain (Figure 4F). Interestingly, we also found that full‐length TEX10 as well as the three abovementioned TEX10 truncations, interacted with RELA (Figure 4G). These results confirmed that TEX10 interacts with RELA and specifically binds to its N‐terminal region.

### 2.5 TEX10 Increases the Nuclear Localization of RELA

**Figure 5:**
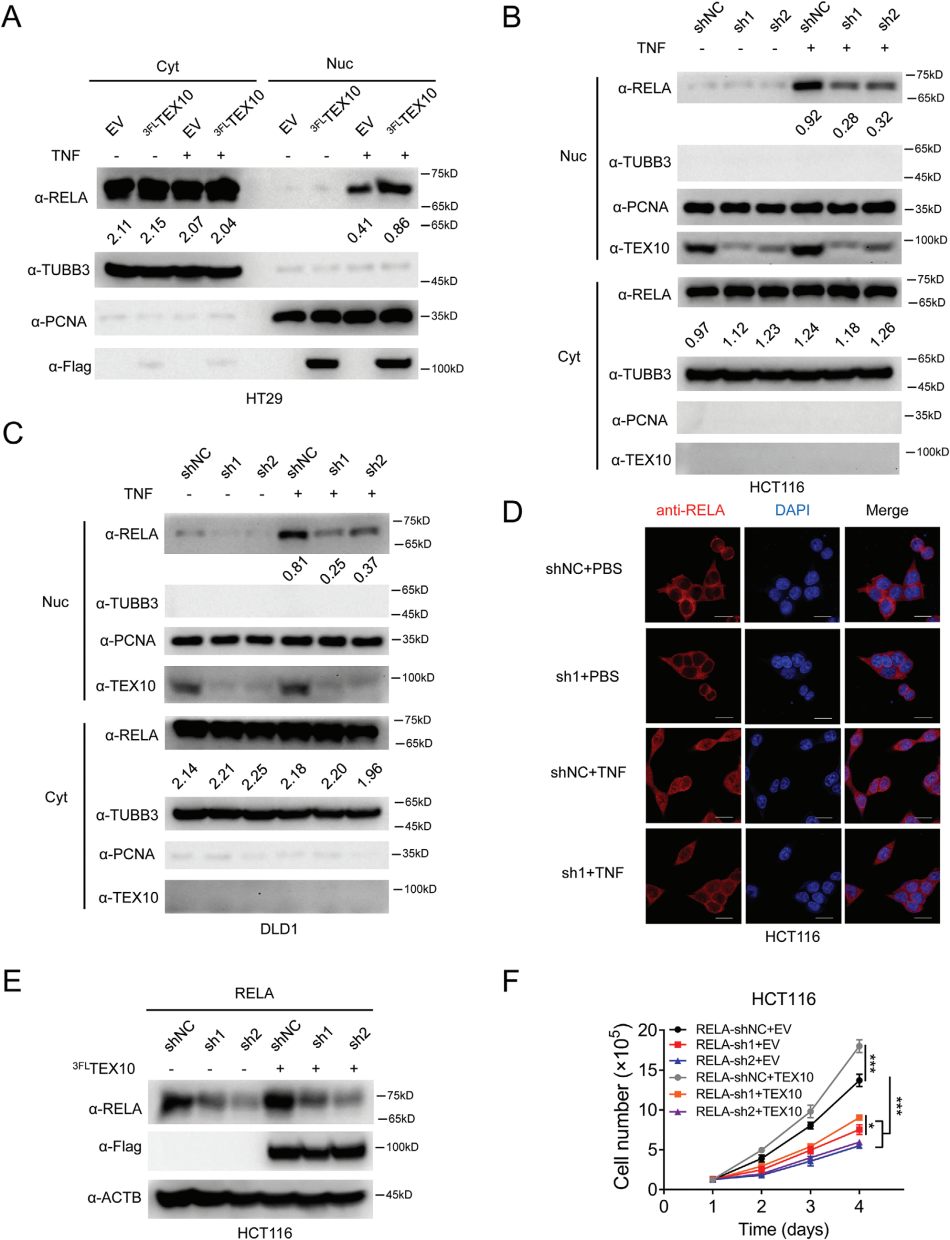
TEX10 increases the nuclear localization of RELA. A) IB analysis of the cytoplasmic (Cyt) and nuclear (Nuc) fractions of HT29 cells infected with 3Flag‐tagged TEX10 (^3FL^TEX10) or EV control. The ratios indicate the RELA intensity normalized to the cytoplasmic TUBB3 or nuclear proliferating cell nuclear antigen (PCNA) intensity. B,C) IB analysis of the Cyt and Nuc fractions of B) HCT116 or C) DLD1 cells infected with TEX10 shRNAs or shNC. The ratios indicate the RELA intensity normalized to that of TUBB3 or PCNA. D) Fluorescence microscopy analysis of HCT116 cells infected with TEX10 shRNA (sh1) or shNC and then stimulated with TNF for 30 min. Nuclei were stained with DAPI (blue). E) IB analysis of RELA, Flag, and ACTB in HCT116 cells infected with RELA shRNAs and then transfected with Flag‐TEX10 or EV. F) Cell count analysis of the growth of the HCT116 cells described in (E). *P < 0.05 and ***P < 0.001 (two‐way ANOVA with Bonferroni's post‐test).

When the NF‐*κ*B pathway is activated, RELA translocates into the nucleus and regulates gene expression.^[^ ^29^ ^]^ We next investigated whether TEX10 could affect this process. Consistent with a previous report,^[^ ^30^ ^]^ TNF treatment increased the nuclear accumulation of RELA, while overexpression of TEX10 further increased the nuclear localization of RELA in HT29 cells (**Figure** **5**A). Additionally, TEX10 knockdown significantly impaired RELA nuclear translocation in HCT116 and DLD1 cells (Figure 5B,C). Immunofluorescence staining showed that TNF‐induced RELA nuclear localization was greatly reduced in TEX10 knockdown cells (Figure 5D). We further ectopically expressed TEX10 in RELA knockdown HCT116 cells (Figure 5E) and found that TEX10 overexpression promoted the proliferation of negative control (shNC) cells, while this effect was abolished in RELA knockdown cells (Figure 5F and Figure S5, Supporting Information). These results suggested that TEX10 increases the nuclear localization of RELA and promotes the proliferation of CRC cells in an RELA‐dependent manner.

### 2.6 TEX10 Regulates the Expression of a Subset of RELA‐Targeted Genes

**Figure 6:**
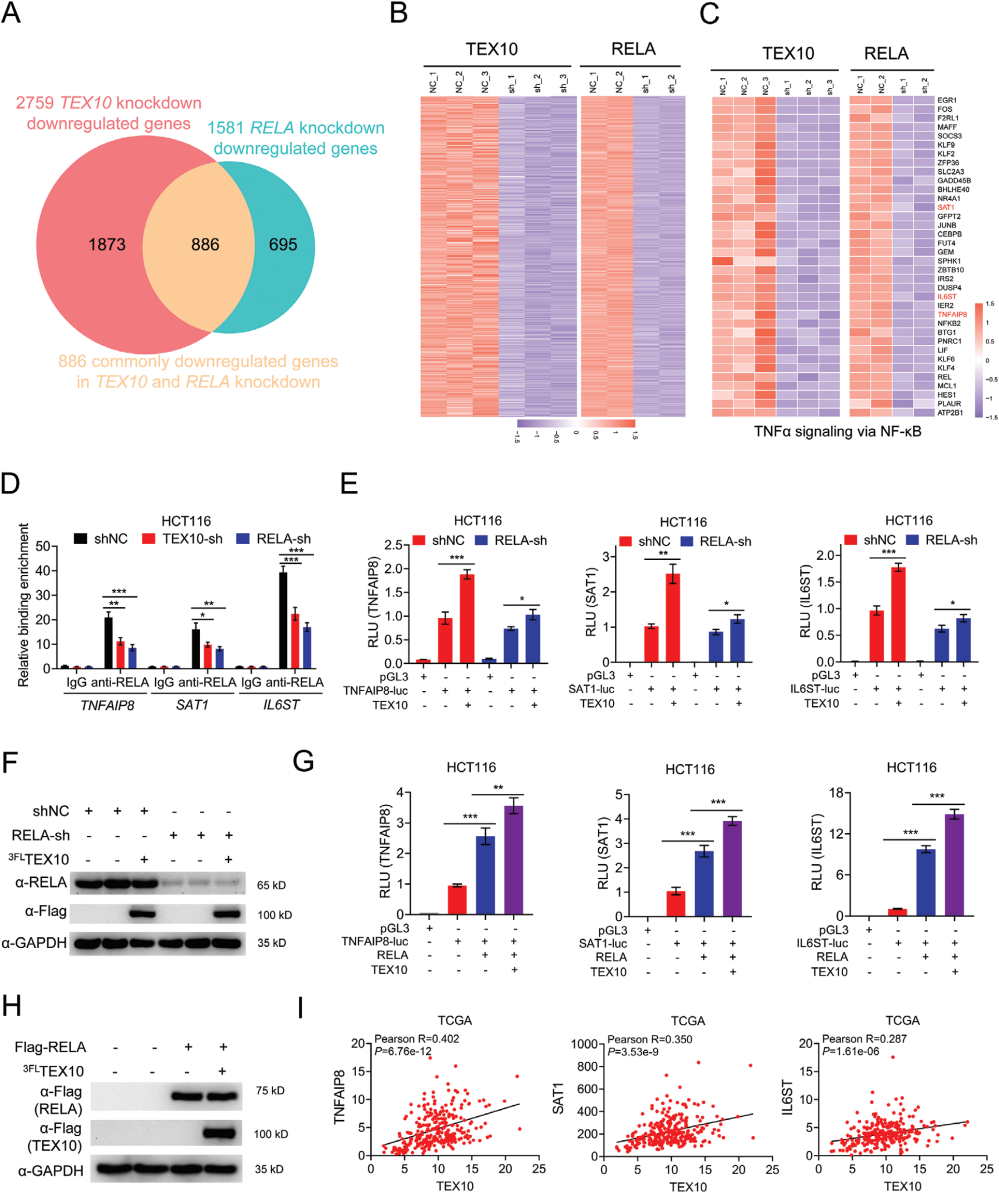
TEX10 regulates the expression of a subset of RELA‐targeted genes. A) RNAseq analysis showed the number of genes downregulated by both TEX10 knockdown and RELA knockdown in HCT116 cells. B) Heatmap of genes downregulated by both TEX10 knockdown and RELA knockdown in HCT116 cells. C) Heatmap of genes downregulated by both TEX10 knockdown and RELA knockdown that are involved in the TNF–NF‐*κ*B pathway in HCT116 cells. D) ChIP qPCR analysis of RELA binding at the TNFAIP8, SAT1, and IL6ST promoters after knockdown of TEX10 or RELA in HCT116 cells. E) Luciferase assay of TNFAIP8, SAT1 and IL6ST promoter‐driven reporters in RELA knockdown and control HCT116 cells transfected with EV or TEX10 for 24 h. F) IB analysis of RELA, Flag, and GAPDH in lysates of the cells described in (E). G) Luciferase assay of TNFAIP8, SAT1, and IL6ST promoter‐driven reporters in HCT116 cells transfected with RELA and EV or TEX10 for 24 h. H) IB analysis of Flag (RELA or TEX10) and GAPDH in the lysates. pGL3, pGL3‐Basic vector; luc, luciferase. I) Correlation analysis of the expression of *TNFAIP8*, *SAT1*, and *IL6ST* with that of *TEX10* in the TCGA dataset. *P < 0.05, **P < 0.01, and ***P < 0.001 (E) two‐tailed Student's *t*‐test or D,G) one‐way ANOVA with Bonferroni's post‐test).

We performed RNAseq analyses and compared the transcriptome of HCT116 cells with RELA knockdown or TEX10 knockdown (Figure S6A, Supporting Information). A total of 2758 and 1581 genes were downregulated in cells with TEX10 knockdown and RELA knockdown, respectively, 886 of which were downregulated in both cell types (**Figure** **6**A,B). Of the 52 NF‐*κ*B target genes targeted by TEX10, 36, including *TNFAIP8*, *SAT1*, and *IL6ST*, were downregulated by both TEX10 knockdown and RELA knockdown (Figures 3B and 6C).

Next, we performed transcription factor binding motif analysis of the promoter regions of these three genes and all of them contained NF‐*κ*B binding sites (Figure S6B, Supporting Information). The chromatin immunoprecipitation (ChIP)‐qPCR assay results indicated that RELA bound to the genomic loci of *TNFAIP8*, *SAT1*, and *IL6ST* and that knockdown of either RELA or TEX10 significantly reduced the level of RELA binding in HCT116 cells (Figure 6D). To further validate the effect of TEX10 on RELA occupancy at these three gene promoters, we constructed promoter‐driven luciferase reporter plasmids. As expected, TEX10 increased the promoter activity of *TNFAIP8*, *SAT1*, and *IL6ST*, but RELA knockdown significantly reduced the function of TEX10 in HCT116 cells (Figure 6E,F). Moreover, the promoters of *TNFAIP8*, *SAT1*, and *IL6ST* were activated by RELA overexpression and this activation was further enhanced by coexpression of TEX10 plasmids (Figure 6G,H).

We analyzed the expression data for CRC in the TCGA database and we found that the expression of *TEX10* was significantly positively correlated with the expression of *TNFAPI8* (Pearson *R* = 0.402, *P *= 6.76e‐12), *SAT1* (Pearson *R* = 0.350, *P *= 3.53e‐9) and *IL6ST* (Pearson *R* = 0.287, *P *= 1.61e‐6) in 270 patients (Figure 6I). These results indicated that TEX10 selectively regulates the expression of a subset of RELA‐targeted genes by promoting RELA occupancy at their promoters.

### 2.7 TEX10 Knockdown Decreases Both the Growth of CRC Cells and Expression of RELA Target Genes In Vivo

**Figure 7:**
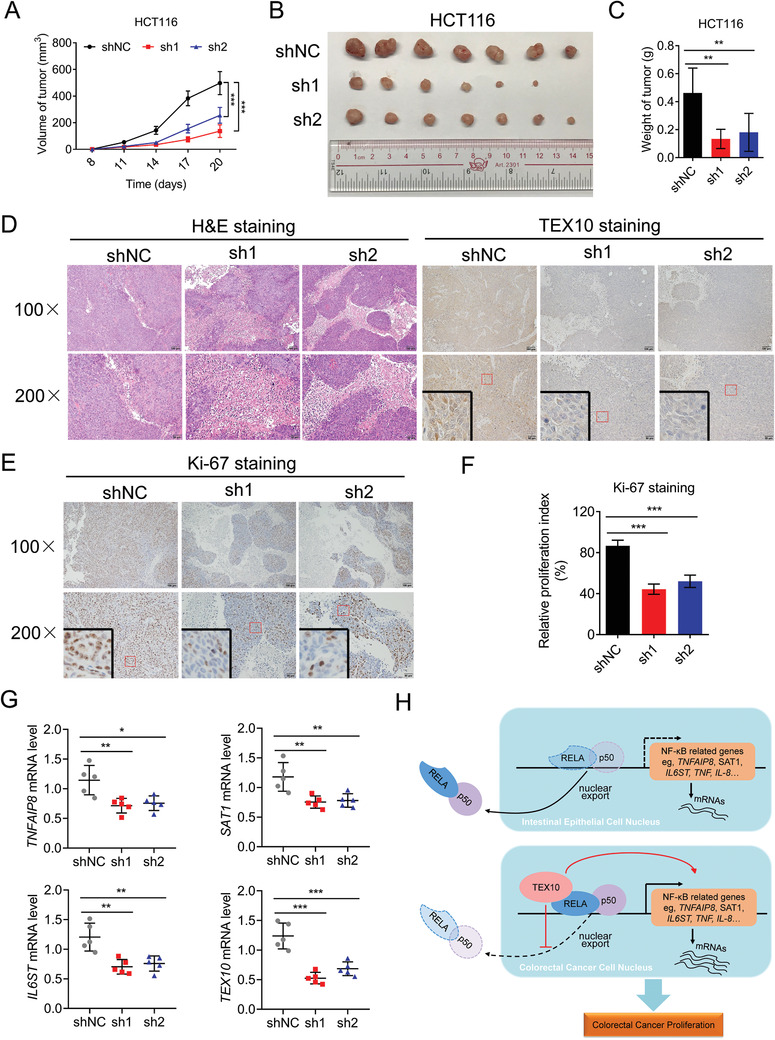
TEX10 knockdown decreases the growth of CRC in vivo. A) Subcutaneous injection of TEX10 knockdown and shNC HCT116 cells into nude mice to establish a xenograft model (*n* = 7). Tumor volumes recorded at the indicated times are shown. B) Tumor images and C) tumor weights on day 20 are shown. D) H&E and TEX10 IHC staining analyses of HCT116 xenograft tumors. E) Ki‐67 IHC staining analyses and F) quantification of the proliferation index (Ki‐67 staining) in HCT116 xenograft tumors. G) qPCR analysis of *TNFAIP8*, *SAT1*, *IL6ST*, and *TEX10* mRNA expression in tumors derived from subcutaneously implanted HCT116 cells infected with TEX10 shRNAs (sh1 and sh2) or shNC. H) A proposed model of the process by which TEX10 promotes proliferation in CRC by increasing NF‐*κ*B activation. *P < 0.05, **P < 0.01, and ***P < 0.001 (one‐way ANOVA with Bonferroni's post‐test).

To determine whether TEX10 was required in established tumors, we injected TEX10 knockdown or control HCT116 cells into the subcutaneous tissue of immunodeficient mice. We measured the growth of subcutaneous tumors and found that tumor growth was significantly slower in the TEX10 knockdown group than that in the control group (**Figure** **7**A). Moreover, the volumes and weights of the tumors from the TEX10 knockdown group were significantly decreased (Figure 7B,C). Hematoxylin and eosin (H&E) and TEX10 IHC staining confirmed that the slow‐growing tumors had reduced expression of TEX10 (Figure 7D). Correspondingly, the TEX10 knockdown group showed a lower cell proliferation index as determined by Ki‐67 IHC staining (Figure 7E,F). Furthermore, in tumors derived from subcutaneously implanted HCT116 cells, *TNFAIP8*, *SAT1*, and *IL6ST* expression were significantly lower in TEX10 knockdown samples (Figure 7G), which also showed decreased in vivo NF‐*κ*B activity, as demonstrated by lower *TNF* and *IL‐8* expression (Figure S7, Supporting Information). These results indicated that TEX10 depletion decreases the growth of CRC cells and the expression of RELA downstream genes in vivo.

## 3 Discussion

Genome‐wide functional screens provide a powerful tool to identify candidate targets for cancer therapy.^[^ ^31^ ^]^ In this study, we applied an shRNA library together with CRC gene profile analyses using published datasets to reveal essential genes in CRC cells, and several genes with known crucial roles in cancer progression and therapy were recurrent. For example, MET receptor (ranked 1st) amplification drives the required resistance of CRC to anti‐EGFR therapy,^[^ ^32^ ^]^ and clinical trials are ongoing to examine EGFR/MET dual inhibition;^[^ ^19^ ^]^ cell‐surface protein TM4SF1 (ranked 25th) promotion of migration and invasion of CRC cells;^[^ ^22^ ^]^ CRC overexpression of the genes PRKDC (ranked 8th) and EXO1 (ranked 14th), both of which are involved in DNA repair and recombination^[^ ^20^, ^33^, ^34^ ^]^ and are required for the survival of cancer cells.^[^ ^35^ ^]^ In addition, the screen revealed TEX10 as a new essential gene in CRC. Although several studies have linked TEX10 to different types of cancer,^[^ ^36^, ^37^ ^]^ its role in gastrointestinal tumors remains unknown. Our in vitro and in vivo experiments demonstrated that TEX10 promotes cell proliferation and functions as an oncogene in CRC. A higher expression level of TEX10 in the cancerous tissues predicts a poor prognosis of CRC patients.

To reveal the mechanism of TEX10 in tumorigenesis, we compared the transcriptome before and after TEX10 silencing. The RNAseq results suggested that TEX10 is associated with several cancer‐related signal pathways, such as TNF signaling, EMT, and hypoxia. TNF is an important cytokine stimulating canonical NF‐*κ*B signaling, which plays an essential role in the regulation of inflammation and has been implicated in the initiation and progression of various tumors, including CRC.^[^ ^13^, ^14^, ^15^ ^]^ The classical NF‐*κ*B is a heterodimer formed by RELA and p50, which shows frequent alterations in expression, posttranslational modifications, or nuclear localization in tumors, resulting in aberrant NF‐*κ*B activation to promote tumor survival, progression, and angiogenesis.^[^ ^38^ ^]^ We observed that TEX10 forms a complex with RELA and thus increases the accumulation of RELA in the nucleus and the enrichment of RELA at the promoter of a subset of its downstream genes including *TNFAIP8*, *SAT1*, and *IL6ST*. All three genes are induced by TNF through NF‐*κ*B binding sites^[^ ^39^, ^40^, ^41^ ^]^ and our results indicated that their promoters could be further activated by TEX10. The inflammatory microenvironments increased the abundance of TNFAIP8, SAT1, and IL6ST in various types of cancer including CRC.^[^ ^42^, ^43^ ^]^ Their elevated expression levels are crucial for the proliferation and survival of CRC cells^[^ ^39^ ^]^ and are associated with a poor prognosis of the patients.^[^ ^43^ ^]^ Moreover, the IL6ST inhibitor bazedoxifene has shown antitumor efficacy in colon cancer,^[^ ^41^ ^]^ prostate cancer,^[^ ^44^ ^]^ and breast cancer,^[^ ^45^ ^]^ suggesting that the TEX10‐NF‐*κ*B‐IL6ST axis is a new target for the precision therapy in CRC.

EMT describes a series of reversible changes in cell phenotype that are involved in several biological processes, such as cancer invasion and metastasis, stem cell maintenance, and embryogenesis, among others.^[^ ^46^ ^]^ The EMT program is believed to be triggered by microenvironments and the inflammation represents such an important factor.^[^ ^47^ ^]^ TEX10 is known to maintain the stemness of ES cells and promote the EMT phenotype of cancer cells via regulating transcription factors such as Oct4, STAT3, or *β*‐catenin.^[^ ^16^, ^36^, ^37^ ^]^ As aberrant activation of NF‐kB contributes to EMT and cancer metastasis,^[^ ^48^ ^]^ it is possibility that TEX10 promotes EMT via an alternative pathway involving the regulation of RELA, which requires further investigation.

In conclusion, our study identified TEX10 as an essential gene in CRC that affects the proliferation of cancer cells and the prognosis of patients. Abnormally elevated expression of TEX10 in cancerous tissues not only induced hyperactivation of the NF‐*κ*B pathway but also promoted the progression of CRC (Figure 7H). Thus, TEX10 may serve as a valuable biomarker and promising therapeutic target in CRC.

## 4 Experimental Section

##### Cell Lines

The normal colon epithelial cell line NCM460 was provided by Prof. Dan Xie (Sun Yat‐sen University Cancer Center, China) and cultured at 37 °C under 5% CO_2_ in Roswell Park Memorial Institute 1640 medium supplemented with 10% fetal bovine serum. Human CRC cell lines HT29, HCT116, HCT15, SW480, SW620, and DLD1 were purchased from American Type Culture Collection (ATCC, Manassas, VA, USA) and cultured according to the instructions of the ATCC. HEK293T cells were in Dulbecco's modified Eagle's medium. The cell lines used in this study have been authenticated.

##### shRNA Library Screening

shRNA library screening and bioinformatics were performed as previously described.^[^ ^17^ ^]^ Briefly, a human pooled lentiviral shRNA library consisting of over 75000 shRNA constructs targeting more than 15 000 human genes was purchased from Sigma‐Aldrich (St. Louis, USA, #SHPH01). HT29 cells were transduced at a multiplicity of infection of 0.4 and were then screened with puromycin (Thermo Fisher Scientific, Waltham, USA, #A1113802) for 4 d. Then total genomic DNA was extracted from day 0 cells and day 4 cells and the shRNA fragments were amplified with primers provided by the library manufacturer. The amplified products were used for second‐generation sequencing using a HiSeq 4000 sequencer (Illumina, San Diego, CA, USA) and the abundance of each shRNA in each group was calculated.

##### Plasmids and Reagents

The expression plasmids for RELA, p50, RELB, p52, and Rel were obtained from the laboratory collection. TEX10 cDNA, TEX10^RES^, and truncations were cloned into the pCDH vector (System Bioscience, Palo Alto, CA, USA). RELA mutants were constructed in pcDNA3.0 vector. For transient transfection, Lipofectamine 2000 (Thermo Fisher Scientific, Waltham, USA, #11668019) was used according to the manufacturer's protocol. TNF (PeproTech, NJ, USA, #96‐300‐01A) and BAY 11‐7085 (Selleck Chemicals, Houston, USA #S2913) were used at a final concentration of 20 ng mL^−1^ and 10 × 10^−6^ m, respectively.

##### Qpcr

Total RNA was extracted with TRIzol reagent (Thermo Fisher Scientific, Waltham, USA, #15596026,) according to the manufacturer's instructions and reverse‐transcribed using One‐step gDNA Removal and cDNA Synthesis Kit (TransGen Biotech, Beijing, China, #AE311). qPCR was performed using the SYBR Green SuperMix (Bio‐Rad, Hercules, USA, #1708884AP) and gene expression was normalized to the control genes glyceraldehyde‐3‐phosphate (GAPDH)/actin beta (ACTB) to calculate relative expression changes. qPCR primers are listed in Table S4 in the Supporting Information.

##### CoIP and IB Analyses

The CoIP and IB analyses were performed as previously described.^[^ ^49^ ^]^ For IB analysis, cells were lysed with radio immunoprecipitation assay (RIPA) lysis buffer (Cell Signaling Technology, Boston, USA, #9806) containing protease inhibitors cocktail (Bimake, Houston, USA, #B14002). After centrifugation, the protein concentration was measured and then equal amounts of lysates were used for IB. For CoIP, cells were collected and lysed in lysis buffer (Cell Signaling Technology, Boston, USA, #9803S) supplemented with protease inhibitors. After centrifugation, supernatants were collected and incubated with appropriate antibodies for 1 h at 4 °C, followed by protein G beads (Santa Cruz Biotechnology, Santa Cruz, USA, #sc‐2002) overnight. After incubation, beads were washed with IP buffer. IB analysis was performed with specific antibodies and secondary antibodies. Antibodies used for IP and IB analyses are listed in Table S5 in the Supporting Information.

##### GST Pull‐Down Assay

GST tagged RELA truncation were cloned into pGEX‐4T‐1 vector and expressed in BL21 chemically competent cells (TransGen Biotech, Beijing, China, #CD901‐01). Myc‐TEX10 (with a T7 promoter) was expressed using TNT T7 Coupled Reticulocyte Lysate Systems according to the manufacturer's instructions (Promega, Madison, USA, #L4611). The in vitro translated Myc‐TEX10 was incubated with GST proteins overnight and analyzed by IB.

##### siRNA Interference

The siRNAs corresponding to the target sequences were synthesized in GenePharma (Suzhou, China). The sequences of siRNAs are listed in Table S6 in the Supporting Information.

##### Stable Cell Line Construction

For establishment of overexpressed stable cells, *TEX10* cDNA was constructed in the pCDH vector. For establishing knockdown cells, shRNAs targeting *TEX10* and *RELA* were cloned into the pLKO.1 vector (Sigma‐Aldrich, St. Louis, MO, USA). For constructing knockout cells, the small guide RNAs (sgRNAs) targeting *TEX10* were cloned into the vector lentiCRIPSPR v2 (Addgene, Cambridge, USA, #52961). Lentiviral expressing plasmid, lentiviral packaging plasmid psPAX.2 (Addgene, Cambridge, USA, #12260) and vesicular stomatitis virus‐glycoprotein (VSV‐G) envelope expressing plasmid pMD2.G (Addgene, Cambridge, USA, #12259) were cotransfected into HEK293T cells. After 48 h, the lentiviruses were used for infecting CRC cells and then screened by puromycin for 3 d. The sequences of shRNAs and sgRNAs are listed in Table S6 in the Supporting Information.

##### Cell Proliferation Assay

For cell growth curves, cell growth was measured by cell count method. For colony‐formation assay, 1 × 10^3^ stable cells were seeded into six‐well plates and cultured for 7–10 d. The cells were then fixed with 4% paraformaldehyde and stained with crystal violet staining solution. Visualize colonies were counted. For EdU staining assay, proliferating cells were detected according to the manufacture's protocol (Beyotime, Shanghai, China, #C0071).

##### Patient Samples

16 CRC tissues were obtained and adjacent normal tissues were matched from Sun Yat‐sen University Cancer Center with the consent of the patients. A total of 129 paraffin‐embedded primary specimens were collected from Sun Yat‐sen University Cancer Center. No patient had received neoadjuvant therapy before surgery. The Ethics Committee of Sun Yat‐sen University Cancer Center approved the study protocols and the informed consent of each patient was required (approval number: GZR2019‐027).

##### RNAseq and Data Analysis

Total RNA was extracted with TRIzol reagent as described above. Total RNA was quality tested and identified to have a sufficiently high quality (RNA integrity number (RIN) ≥ 9.5) for construction of sequencing libraries. After mRNA capture, mRNA fragmentation, reverse transcription, terminal repair, linker ligation and PCR amplification, second‐generation sequencing was performed according to Illumina's standard protocol. Quality control (QC) of raw reads from all samples was performed using FastQC (v0.11.2) software. Spliced trans alignment to a reference (STAR) (v2.4.2a) software was used to perform sequence alignment on the clean reads of each sample. GSEA of the sequencing results for TEX10‐shNC and TEX10‐sh cells was performed. The R programming language was used to calculate FDR values and construct heatmaps.

##### Assay of Luciferase Activity

Reporter plasmid, Prl‐TK plasmid and the expression plasmids were cotransfected into cells for 24 h. Luciferase activity was detected with the Dual‐Luciferase Reporter Assay System according to the manufacturer's instructions (Promega, Madison, USA, #E1910). TNFAIP8 and SAT1 promoters (1500 bp upstream of the start site) and IL6ST promoter (1000 bp upstream of the start site plus 500 bp downstream of the start site) were constructed in pGL3 basic vector.

##### Bioinformatics Analysis of the GEO and TCGA Data

The expression data in colorectal cancer and normal tissues were extracted from GEO dataset GSE8671^[^ ^18^ ^]^ and the OS data of CRC patients were extracted from GSE12945,^[^ ^50^ ^]^ GSE17536, and GSE17537.^[^ ^51^, ^52^, ^53^ ^]^ The TEX10 expression in various tumors from TCGA data was analyzed on the Gene expression prolifiling interactive analysis (GEPIA) website^[^ ^54^ ^]^ (http://gepia.cancer-pku.cn/) and the normal data were from match TCGA normal and the Genotype‐Tissue Expression (GTEx) data. Expression profiles of *RELA* and *NFKB2* across human normal tissues were analyzed on the G E‐mini website^[^ ^28^ ^]^ (http://gemini.cancer-pku.cn/). Correlation analysis of gene expression in 270 patients was extracted from TCGA database.

##### Immunofluorescence (IF) Assay

IF assay was performed as previously described.^[^ ^49^ ^]^ Briefly, cells were grown on coverslips (NEST Biotechnology Co.LTD. (NEST), Wuxi, China, #801009) and stimulated with TNF after 12 h. After fixation (4% paraformaldehyde), permeabilization (0.2% Triton X‐100), and blocking (5% bovine serum albumin), cells were incubated with mouse anti‐RELA antibody (Abclonal, Wuhan, China, #A10609) for 2 h followed by TetramethylRodaminesoThioCyanate (TRITC)‐conjugated antimouse secondary antibody for 1 h. After staining the nucleus with 4,6‐diamino‐2‐phenyl indole (DAPI) (KeyGEN Biotechnology, Nanjing, China, #KGA215), images were observed with a laser confocal fluorescence microscopy (Zeiss, LSM880, Oberkochen, Germany).

##### ChIP Assay

ChIP assay was performed according to the manufacturer's protocol (Cell Signaling Technology, Boston, USA, #9005). Input DNA and immunoprecipitated DNA were detected by qPCR and the data were normalized to the input control. ChIP qPCR primers are listed in Table S7 in the Supporting Information.

##### Animal Experiments

All animal experiments were conducted in accord with the National Institute of Health Guide for the Care and Use of Laboratory Animals, maintaining the animals under specific‐pathogen‐free conditions. The experimental procedures were approved by Animal welfare and Ethics Committee of Sun Yat‐sen University Cancer Center (approval number: L102012019000P). 4‐ to 5‐week‐old female BABL/c nude mice were purchased from Beijing Vital River Laboratory Animal Technology Co (Beijing, China) and randomly assigned to different groups. For tumor proliferation assay, 1 × 10^6^ stable HCT116 cells were subcutaneously injected into mice. Tumor volume was measured every 3 d after 1 week of injection. Tumor volume was calculated using the following formula: volume = 0.52 × weight^2^ × length. After 20 d of injection, the mice were sacrificed and the tumors removed and weighed.

##### IHC Staining and Prognostic Value Analysis

For paraffin‐embedded tissue sections, deparaffinization, rehydration, antigen retrieval, and endogenous peroxidase inactivation (3% H_2_O_2_) were performed. After blocking, the slides were incubated with rabbit pAb against TEX10 (Affinity Biosciences, Cincinnati, OH, USA, #DF12486, 1:100 for IHC); mouse mAb against Ki‐67 (BD Biosciences, Franklin, USA, #550609, 1:100 for IHC) at 4 °C overnight. Subsequently, they were incubated with a horseradish peroxidase‐conjugated secondary antibody and visualized with antirat/rabbit Universal Immunohistochemical Detection Kit (Proteintech Group, Chicago, USA, #KIHC‐5). The nucleus was counterstained with hematoxylin. The TEX10 levels were independently evaluated by two pathologists. TEX10 is located in the nucleus and the staining intensity score of TEX10 was divided into four classes, namely 0, absent; 1, weak; 2, moderate; and 3, strong. The final score was calculated by multiplying the positive cell percentage score by the average staining intensity. The patients were categorized into two groups based on TEX10 level (high or low) and the cutoff value was deduced using a receiver operating characteristic curve.

##### Statistical Analysis

All in vitro experiments were repeated at least three times and in vivo experiments were repeated twice. Data were analyzed using SPSS software and generated with GraphPad Prism 8. Statistical analysis was performed with Student's *t*‐test between two independent groups and one‐way analysis of variance (ANOVA) with Bonferroni's post‐test or two‐way ANOVA with Bonferroni's post‐test among multiple groups. Survival curves were analyzed using the Kaplan–Meier method and the log‐rank test. The Cox proportional hazards model was used for analyzing associations of variables with survival. *P* < 0.05 was considered statistically significant and *P* values were indicated by asterisks as followed: * *P* < 0.05, ** *P* < 0.01, *** *P* < 0.01, and n.s. = nonsignificant.

##### Data Availability

RNAseq data have been submitted to the GEO with accession number GSE147334. Raw data of this study have been deposited to the Research Data Deposit database (http://www.researchdata.org.cn), with the Approval Number as RDDB2020000881.

## Conflict of Interest

The authors declare no conflict of interest.

## Author Contributions

Z.W. performed most of the experiments and analyses. Z.W. and C.Y. performed the mouse experiments. R.G. performed the RNA interference (RNAi) screening. C.S. provided technical assistance. Z.W. and G.K. extracted RNA and proteins from tumors and adjacent normal tissues in patients with colorectal cancer. S.C. conceived the study. S.C. and Z.W. wrote the paper.

## Supporting information

Supporting Information

Supporting Table 1
