# Supplementary material for: RNAi Screening Identifies that TEX10 Promotes the Proliferation of Colorectal Cancer Cells by Increasing NF‐κB Activation
Source: Adv Sci (Weinh). 2020 Jul 7;7(17):2000593. doi: 10.1002/advs.202000593 (PMC7507032; doi:10.1002/advs.202000593)
Supplement: Supplementary file 1 — Supporting Information [file ADVS-7-2000593-s001.pdf]

Supporting Information

RNAi Screening Identifies TEX10 Promoting the Proliferation of Colorectal Cancer by Increasing NF-κB Activation

*Ziyang Wang, Chunjie Sheng, Guangyan Kan, Chen Yao, Rong Geng, and Shuai Chen**

This supporting information includes:

1) Seven supplementary figures

2) Seven supplementary tables

Supplementary Figures

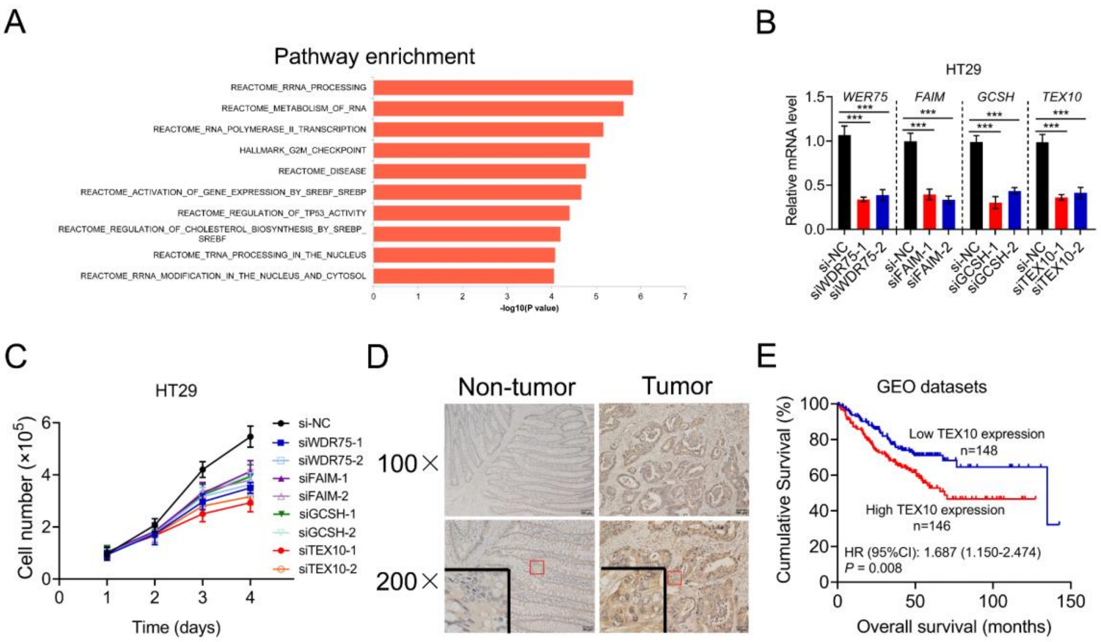

Figure S1. Screening determines that TEX10 promotes CRC proliferation. (**A**) Pathway enrichment of screening 149 genes. (**B**) qPCR analysis of siRNAs knockdown efficiency in HT29 cells. (**C**) Cell count analysis of the growth of siRNAs knockdown cells in HT29 cells. (**D**) IHC staining of TEX10 in CRC and adjacent non-tumor tissues. (**E**) Kaplan-Meier analysis of OS according to TEX10 mRNA levels in GEO datasets GSE12945, GSE17536 and GSE17537. $^{***}P < 0.001$ (one-way ANOVA with Bonferroni's post-test (B) or log-rank test (E)).

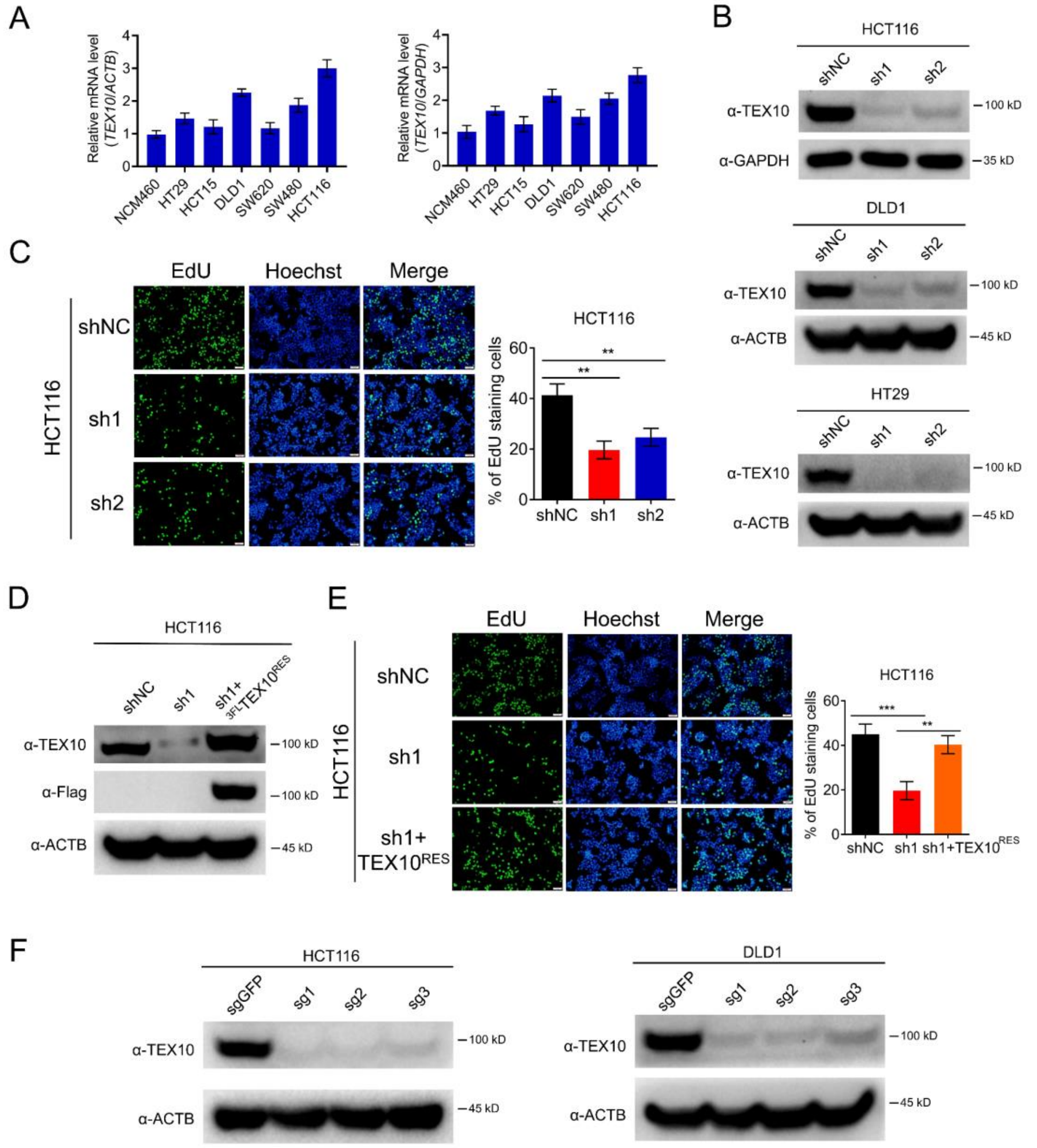

Figure S2. TEX10 knockdown decreases the growth of CRC cells *in vitro*. (**A**) qPCR analysis of *TEX10* mRNA expression in CRC cell lines. (**B**) IB analysis of the TEX10 knockdown efficiency in HCT116, DLD1 and HT29 cells. (**C**) EdU staining analysis of the proliferation of shNC and TEX10 knockdown HCT116 cells. (**D**, **E**) IB analysis (**D**) and EdU staining analysis (**E**) of TEX10 protein expression in HCT116 shNC, TEX10-sh1 and TEX10-sh1 knockdown cells reconstituted with $TEX10^{RES}$. (**F**) IB analysis of the TEX10 knockout efficiency in HCT116 and DLD1 cells. $^{**}P < 0.01$, and $^{***}P < 0.001$ (one-way ANOVA with Bonferroni's post-test).

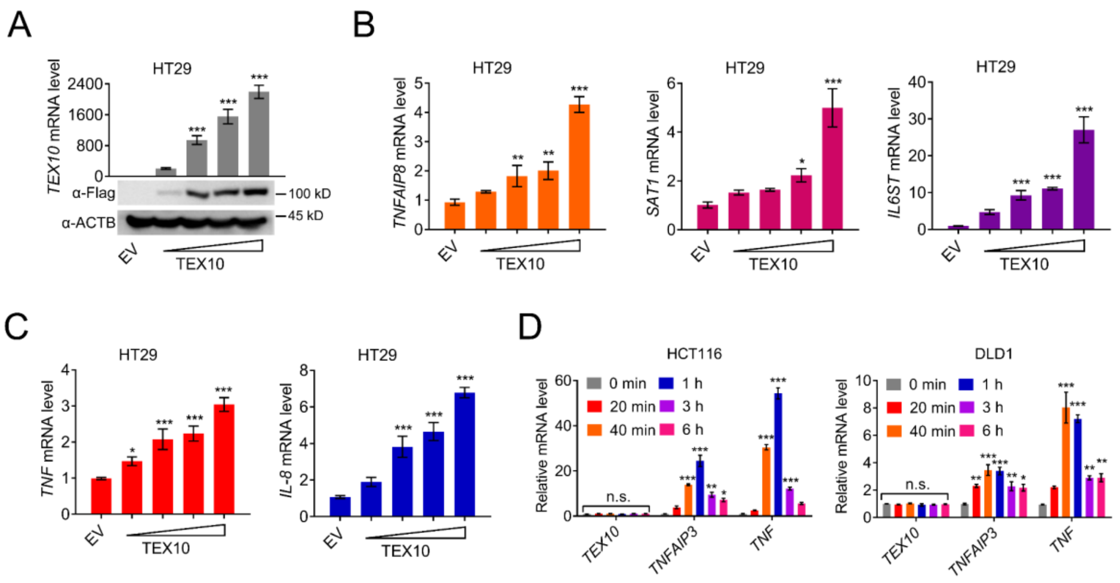

Figure S3. TEX10 affects NF-κB activity. (**A**) qPCR and IB analyses of TEX10 expression in HT29 cells transfected with EV and different doses of TEX10 (500, 1000, 1500 and 2000 ng). (**B**, **C**) qPCR analysis of *TNFAIP8*, *SAT1* and *IL6ST* mRNA levels (**B**) or *TNF* and *IL-8* mRNA levels (**C**) in the HT29 cells indicated in (**A**). (**D**) qPCR analysis of the mRNA expression of the indicated genes in HCT116 or DLD1 cells stimulated with TNF for different times. *$P < 0.05$, **$P < 0.01$, and ***$P < 0.001$ (one-way ANOVA with Bonferroni's post-test).

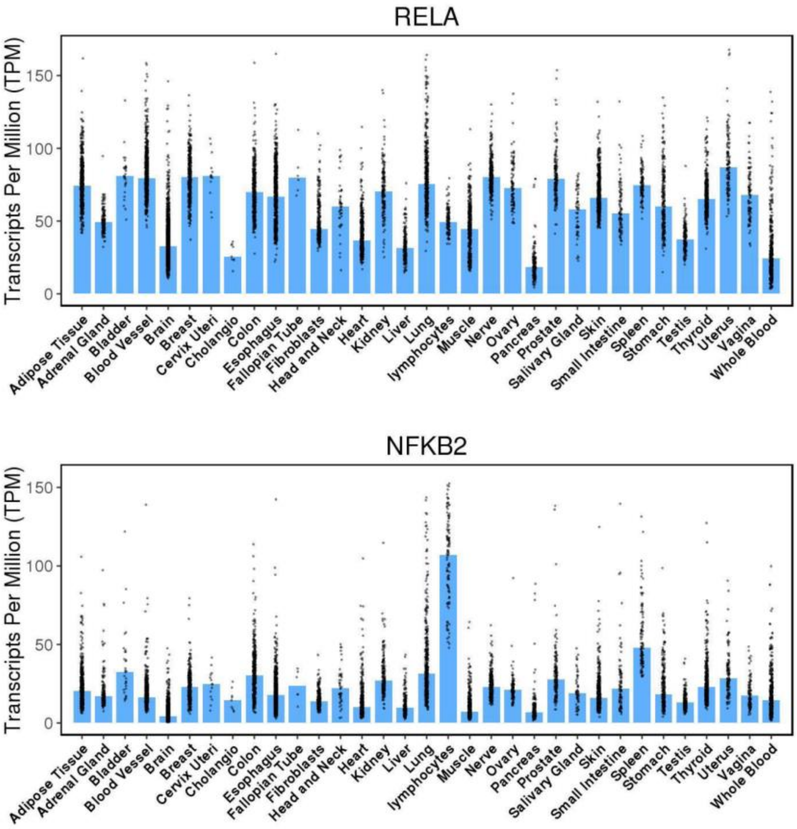

Figure S4. Expression profiles of *RELA* and *NFKB2* across human normal tissues as identified through the GE-mini website.

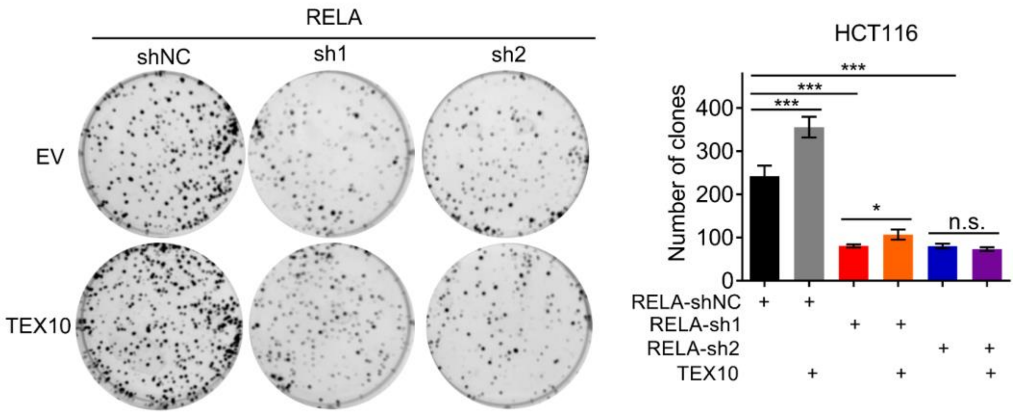

Figure S5. TEX10 functions through RELA. Colony formation assay of the growth of HCT116 cells infected with RELA shRNAs (sh1 and sh2) and then transfected with Flag-TEX10 or EV. **P* < 0.05, ****P* < 0.001, and n.s. = non-significant (two-way ANOVA with Bonferroni's post-test).

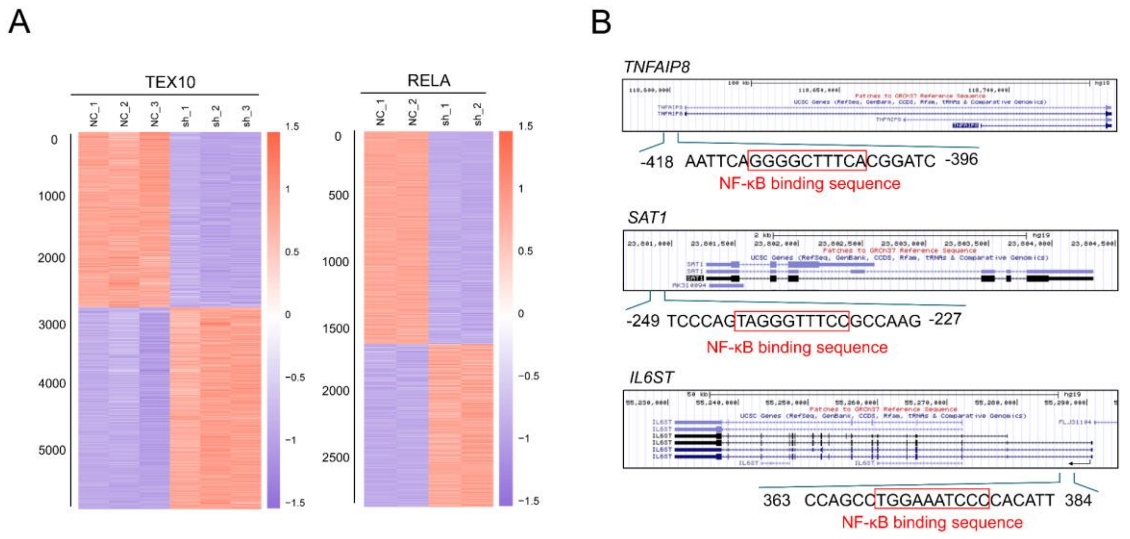

Figure S6. TEX10 regulates the expression of a subset of RELA-targeted genes. (**A**) Heatmap of differentially expressed genes (FDR < 0.01) in TEX10 or RELA knockdown HCT116 cells compared with shNC cells. (**B**) NF-κB binding sequences in the TNFAIP8, SAT1 and IL6ST promoters.

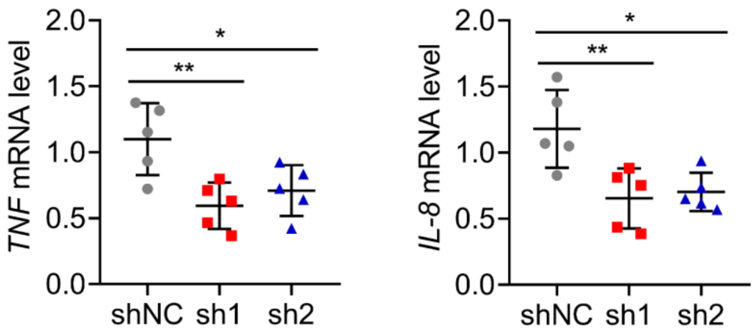

Figure S7. TEX10 knockdown affects NF-κB activity *in vivo*. qPCR analysis of *TNF* and *IL-8* mRNA expression in tumors derived from subcutaneously implanted HCT116 cells infected with TEX10 shRNAs (sh1 and sh2) or shNC. *$P < 0.05$, and **$P < 0.01$ (one-way ANOVA with Bonferroni's post-test).

Supplementary Tables

Table S2. Multivariate Cox proportional hazards regression analysis for OS and PFS in CRC patients

Variable	OS		PFS	
	HR (95% CI)	*P* value	HR (95% CI)	*P* value
Age (year;<55 vs >55)	0.7220 (0.4076-1.279)	0.2731	0.6861 (0.3872-1.216)	0.1841
Gender (male vs female)	1.015 (0.5687-1.810)	0.9955	0.9941 (0.5577-1.772)	0.905
Tumor location (colon vs rectum)	0.8558 (0.4827-1.517)	0.4581	0.8401 (0.4737-1.490)	0.4319
Tumor size (cm; <5 vs >5)	1.560 (0.8747-2.783)	0.098	1.546 (0.8662-2.759)	0.1086
Tumor invasive depth (T1-2 vs T3-4)	3.016 (1.511-6.019)	0.0158*	3.057 (1.535-6.086)	0.0134*
Lymph node status (<1vs ≥1)	3.285 (1.840-5.866)	<0.0001*	3.323 (1.860-5.936)	<0.0001*
Distant metastasis (No vs Yes)	10.86 (4.364-27.03)	<0.0001*	9.912 (4.111-23.90)	<0.0001*
Stage (I-II vs III-VI)	8.198 (4.585-14.66)	<0.0001*	8.310 (4.644-14.87)	<0.0001*
Preoperative CEA (ng/ml; <5 vs ≥5)	1.461 (0.7958-2.682)	0.1172	1.449 (0.7896-2.660)	0.1381
Preoperative CA199(ng/ml; <35 vs ≥35)	2.952 (1.129-7.718)	0.0037*	3.122 (1.177-8.283)	0.0021*
TEX10 (low vs high)	2.368 (1.316-4.264)	0.003*	2.564 (1.416-4.643)	0.001*

Abbreviations: OS, overall survival; PFS, progression-free survival; HR, hazard ratio; CI, Confidence interval; CEA, carcino-embryonic antigen; CA199, carbohydrate antigen 19-9.

*Statistically significant: $P < 0.05$.

Table S3. Clinicopathological characteristics and their correlation with TEX10 expression in 129 CRC patients

Parameters	N (%)	TEX10 low N=74	TEX10 high N= 55	*P*
Age				
<56	65 (50.4%)	37 (28.7%)	28 (21.7%)	
≥56	64 (49.6%)	37 (28.7%)	27 (20.9%)	0.44
Gender				
Male	75 (58.1)	47 (36.4%)	28 (21.7)	
Female	54 (41.9)	27 (20.9%)	27 (20.9%)	0.72
Tumor size(cm)				
<5	50 (38.8%)	29 (22.5%)	21 (16.3%)	
≥5	79 (61.2)	45 (34.9)	34 (26.4%)	0.75
Tumor invasive depth				
T1-T2	23 (17.8%)	14 (10.9%)	9 (7.0%)	
T3-T4	106 (82.2%)	60 (46.5%)	46 (35.7%)	0.56
Tumor location				
Colon	64 (49.6)	37 (28.7%)	28 (21.7%)	
Rectum	65 (50.4%)	37 (28.7%)	27 (20.9%)	0.96
Lymph node status				
<1	66 (51.2%)	43 (33.3%)	23 (17.8%)	
≥1	63 (48.8%)	31 (24.0%)	32 (24.8%)	0.001*
AJCC/TNM stage				
I-II	62 (48.1%)	41 (31.8%)	21 (16.3%)	
III-IV	67 (51.9%)	33 (25.6%)	34 (26.4%)	0.0003*
Distant metastasis				
No metastasis	100 (77.5%)	59 (45.7%)	41 (31.8)	
Metastasis	29 (22.5%)	15 (11.6%)	14 (10.9%)	0.02*
Preoperative CA199 (ng/ml)				
≤5	61 (47.3%)	35 (27.1%)	26 (22.5)	
>5	60 (46.5%)	34 (26.4%)	26 (22.5)	0.44
Preoperative CA199 (ng/ml)				
≤35	60 (46.5%)	33 (25.6%)	27 (20.9%)	
>35	24 (18.6%)	11 (8.5%)	13 (10.1%)	0.26

Abbreviations: CEA, carcino-embryonic antigen; CA199, carbohydrate antigen 19-9.

*Statistically significant: $P < 0.05$.

Table S4. Quantitative real-time PCR primers

Gene name	Forward (5'-3')	Reverse (5'-3')
ACTB	GCGTGACATTAAGGAGAAG	GAAGGAAGGCTGGAAGAG
GAPDH	GAAGGTGAAGGTCGGAGTC	GAAGATGGTGATGGGATTTC
WDR75	AACACAGGCAACAGCAGGATG	GCACAGGAAAGCAGCAGATGG
FAIM	CTGTTGTGCTATAATCATCTCTTG	CATCTACATATACTACTCGTTTGC
GCSH	CTGGACCCGCTCTGCTCTC	CATCTCCCAACGCTTCCTGTG
TEX10	GCCAACGACCAGCAACACATC	CACTCAGTCCTCCAACCAGATACC
TNFAIP8	AAGTAGTGAGGTGCTGGATGAG	GCTTGATGACTGTCTTGATGAGG
SAT1	TATTGTATCTTGAGGACTTCTTC	GTTCATTCCATTCTGCTACC
IL6ST	ACACCAAGTTCCGTCAGTC	ATACCATCACCGCCATCTAC
TNF	AGTGAAGTGCTGGCAACCAC	GAGGAAGGCCTAAGGTCCAC
IL-8	CTGCGCCAACACAGAAATTAT	CATCTGGCAACCCTACAACAG

Table S5. Antibodies used for IP and IB analyses

Antibody	Source	Dilution	Company
TEX10	Mouse mAb (sc-398384)	1:200 for IP<br>1:1000 for IB	Santa Cruz Biotechnology, Santa Cruz, USA
RELA	Rabbit mAb (A10609)	1:1000 for IB	Abclonal, Wuhan, China
Flga	Mouse mAb (F1804)	1:400 for IP<br>1:1000 for IB	Sigma Aldrich, St. Louis, USA
Myc	Mouse mAb (RM1003)	1:400 for IP<br>1:1000 for IB	Beijing Ray Antibody Biotech, Beijing, China
ACTB (β-actin)	Mouse mAb (60008-1-Ig)	1:2000 for IB	Proteintech Group, Chicago, USA
GAPDH	Mouse mAb (RM2002)	1:2000 for IB	Beijing Ray Antibody Biotech, Beijing, China
p-RELA (Ser536)	Rabbit mAb (3033)	1:1000 for IB	Cell Signaling Technology, Boston, USA
p-IκBα (Ser32/36)	Mouse mAb (9246)	1:1000 for IB	Cell Signaling Technology, Boston, USA
IκBα	Mouse mAb (4814)	1:1000 for IB	Cell Signaling Technology, Boston, USA
TUBB3 (β-Tubulin)	Mouse mAb (RM2003)	1:2000 for IB	Beijing Ray Antibody Biotech, Beijing, China

PCNA	Rabbit pAb (10205-2-AP)	1:2000 for IB	Proteintech Group, Chicago, USA
Secondary antibodies	HRP-conjugated antibodies to mouse (115-035-003)	1:5000 for IB	Jackson ImmunoResearch, Philadelphia, USA
Secondary antibodies	HRP-conjugated antibodies to rabbit (111-035-003)	1:5000 for IB	Jackson ImmunoResearch, Philadelphia, USA
Secondary antibodies	HRP-conjugated antibodies to mouse IgG, Fcγ fragment specific (115-035-071)	1:5000 for IB	Jackson ImmunoResearch, Philadelphia, USA
Secondary antibodies	HRP-conjugated antibodies to mouse IgG, light chain specific (115-035-174)	1:5000 for IB	Jackson ImmunoResearch, Philadelphia, USA
Secondary antibodies	HRP-conjugated antibodies to rabbit IgG, Fc fragment specific (111-035-046)	1:5000 for IB	Jackson ImmunoResearch, Philadelphia, USA
Secondary antibodies	HRP-conjugated antibodies to rabbit IgG, light chain specific (211-032-171).	1:5000 for IB	Jackson ImmunoResearch, Philadelphia, USA

Table S6. The sequences of siRNAs, shRNAs and sgRNAs

Identifier	Sequences (5'-3')
siWDR75-1	GCUGCAAUCUCUCAGUCUUTT
siWDR75-2	GCAUUACAGCUCUCUGUUUTT
siFAIM-1	CCACCAAUACUUGGGUAUUTT
siFAIM-2	GCUGUUUGGGAUGUUGCUUTT
siGCSH-1	GGCAUUGGAACAGUGGGAATT
siGCSH-2	GCCAAGAUCUGUCACCUUUTT
siTEX10-1	GCUCUAGGCUAAAUAGUAATT
siTEX10-2	GCUACUGCCCUCCGAAUUUTT
TEX10-sh1	GCGTTTGGTTCAGCTTGTATA
TEX10-sh2	AGATTCAGATATCGTAGTAAA
TEX10-sg1	CCTACTGGATCCATCTGCCA
TEX10-sg2	ATTGTAGGGACAAGCTGTGG
TEX10-sg3	TCAGTTCACAGTTCAGGCTA
RELA-sh1	GCCTTAATAGTAGGGTAAGTT
RELA-sh2	CGGATTGAGGAGAAACGTAAA

Table S7. ChIP PCR primers

Gene name	Forward (5'-3')	Reverse (5'-3')
TNFAIP8	CAAATGGAAACCAGGAATCGG	GCGTGAATGTTAGTGTCTGC
SAT1	GGGAATTACCTTCTTTCATTTGC	CCCTCTAGTGGCGTTTCG
IL6ST	GTTTCGGACGGGCTATCTG	GGCTGGGCTGACAAGTTC
